# Association of air pollution exposure and increased coronary artery disease risk: the modifying effect of genetic susceptibility

**DOI:** 10.1186/s12940-023-01038-y

**Published:** 2023-12-08

**Authors:** Zuqiang Fu, Yuanyuan Ma, Changjie Yang, Qian Liu, Jingjia Liang, Zhenkun Weng, Wenxiang Li, Shijie Zhou, Xiu Chen, Jin Xu, Cheng Xu, Tao Huang, Yong Zhou, Aihua Gu

**Affiliations:** 1 State Key Laboratory of Reproductive Medicine and Offspring Health, School of Public Health, Nanjing Medical University, 101 Longmian Avenue, Nanjing, 211166 China; 2 Collaborative Innovation Center for Cardiovascular Disease Translational Medicine, Nanjing Medical University, Nanjing, China; 3 Department of Toxicology, Center for Global Health, Nanjing Medical University, Nanjing, China; 4 School of Public Health, Southeast University, 101 Longmian Avenue, Nanjing, 211166 China; 5 Department of Maternal, Child, and Adolescent Health, School of Public Health, Nanjing Medical University, Nanjing, China; 6 Department of Epidemiology and Biostatistics, School of Public Health, Peking University, 38 Xueyuan Road, Beijing, 100191 China; 7 CAS Key Laboratory of Tissue Microenvironment and Tumor, Shanghai Institute of Nutrition and Health, Shanghai Institutes for Biological Sciences, University of Chinese Academy of Sciences, Chinese Academy of Sciences, No. 320 Yueyang Road, Shanghai, 200031 China

**Keywords:** Air pollution, Coronary artery disease, Polygenic risk score, Gene‒environment interaction

## Abstract

**Background:**

Both genetic factors and air pollution are risk factors for coronary artery disease (CAD), but their combined effects on CAD are uncertain. The study aimed to comprehensively investigate their separate, combined and interaction effects on the onset of CAD.

**Methods:**

We utilized data from the UK Biobank with a recruitment of 487,507 participants who were free of CAD at baseline from 2006 to 2010. We explored the separate, combined effect or interaction association among genetic factors, air pollution and CAD with the polygenic risk score (PRS) and Cox proportional hazard models.

**Results:**

The hazard ratios (HRs) [95% confidence interval (CI)] of CAD for 10-µg/m^3^ increases in PM_2.5_, NO_2_ and NO_x_ concentrations were 1.25 (1.09, 1.44), 1.03 (1.01, 1.05) and 1.01 (1.00, 1.02), respectively. Participants with high PRS and air pollution exposure had a higher risk of CAD than those with the low genetic risk and low air pollution exposure, and the HRs (95% CI) of CAD in the PM_2.5_, PM_10_, NO_2_ and NO_x_ high joint exposure groups were 1.56 (1.48, 1.64), 1.55(1.48, 1.63), 1.57 (1.49, 1.65), and 1.57 (1.49, 1.65), respectively. Air pollution and genetic factors exerted significant additive effects on the development of CAD (relative excess risk due to the interaction [RERI]: 0.12 (0.05, 0.19) for PM_2.5_, 0.17 (0.10, 0.24) for PM_10_, 0.14 (0.07, 0.21) for NO_2_, and 0.17 (0.10, 0.24) for NO_x_; attributable proportion due to the interaction [AP]: 0.09 (0.04, 0.14) for PM_2.5_, 0.12 (0.07, 0.18) for PM_10_, 0.11 (0.06, 0.16) for NO_2_, and 0.13 (0.08, 0.18) for NO_x_).

**Conclusion:**

Exposure to air pollution was significantly related to an increased CAD risk, which could be further strengthened by CAD gene susceptibility. Additionally, there were positive additive interactions between genetic factors and air pollution on the onset of CAD. This can provide a more comprehensive, precise and individualized scientific basis for the risk assessment, prevention and control of CAD.

**Supplementary Information:**

The online version contains supplementary material available at 10.1186/s12940-023-01038-y.

## Background

Coronary artery disease (CAD), also called coronary heart disease (CHD), is the leading cause of multiple metabolic diseases and mortality [1–3]. To date, CAD is still incurable, and its pathogenesis is not clear [4]. Therefore, CAD prevention becomes particularly important, especially the identification and risk assessment of risk factors, which are the first steps in the prevention of CAD [1]. In addition to some common risk factors, such as age, sex and race, recent studies have more consistently shown that air pollution also leads to cardiovascular disease (CVD) [5–8].

Moreover, the limited previous studies involving the relationship between air pollution and CAD risk were mainly concentrated on certain subclinical or clinical disorders of CAD, such as coronary vasomotor abnormalities [9], coronary artery calcium [10, 11], acute myocardial infarction [12] and coronary plaques [13]. Other studies mainly considered the risk of near-roadway air pollution (NRAP) on CAD morbidity [14, 15]. However, national prospective population studies assessing the relationship between ambient air pollution exposure and CAD risk remain scarce.

Additionally, it is well accepted that both hereditary susceptibility and air pollution exposure contribute to the risk of CAD, but neglect of their combined or interaction effects made the results biased [1]. Recently, increasing evidence has reported that genetic susceptibility could interact with environmental factors to affect the onset of cardiometabolic diseases [16, 17], including CVDs [18, 19]. However, whether the air pollution exposure alters the association between genetic factors and CAD remains unknown.

Therefore, by means of the polygenic risk score (PRS) [20, 21], we applied data from the UK Biobank with comprehensive information on common baseline characteristics, exposure factors and outcomes to evaluate the separate, combined effect or interaction association between genetic factors and air pollutants, including particulate matter with diameters ranging from ≤ 2.5–≤10 μm (PM_2.5_, PM_2.5−10_ and PM_10_), nitrogen dioxide (NO_2_), and nitrogen oxides (NO_x_), and the risk of CAD.

## Methods

### Study cohort and data access

The current study utilized data from the UK Biobank, a national multicentre prospective cohort study [22]. Briefly, ~ 0.5 million residents aged 40–69 years between 2006 and 2010 were recruited from the UK National Health Service and living < 25 miles from 1 of the 22 study assessment centres across the UK (England, Wales and Scotland). The baseline summary characteristics can be viewed at the website of UK Biobank (https://biobank.ndph.ox.ac.uk/showcase/). The UKB was approved by the North West Multi-Center Research Ethics Committee, and all participants provided informed consent forms.

All eligible researchers could submit the application for data access. The detailed access procedure can be found at UK Biobank’s website (http://www.ukbiobank.ac.uk/register-apply/).

### Air pollution exposure assessment

Average exposure to air pollutants, including PM_2.5_, PM_2.5−10_, PM_10,_ NO_2_ and NO_x_, was assessed in the UK Biobank study under strict measures and internationally recognized standards [23, 24]. Details can be viewed in the Supplementary Materials.

### CAD ascertainment

The outcome of this study was CAD, which was defined with the 9th /10th Revision of International Classification of Diseases (ICD). In the UK Biobank, CAD was determined with the Office of Population Censuses and Surveys’ Classification of Interventions and Procedures, self-reported diagnoses and Hospital Episode Statistics data, as previously described [25], and the corresponding UK Biobank codes are provided in Table S1. This definition of CAD includes myocardial infarction and its related sequelae.

### Calculation of covariates

We included multiple covariates including baseline characteristics such as age and sex, and health-related outcomes. The detailed contents about variable assessment and data access code are provided in the Supplementary Materials.

### PRS contraction

We used the PRS to calculate the accumulative effects of multiple genetic variations, and the detailed definition and formula are presented in the Supplementary Materials. The present PRS utilized data from the largest available CAD genome-wide association meta-analysis without the UK Biobank population [26], with 44 single nucleotide polymorphisms (SNPs). Forty SNPs were finally available in the UK Biobank imputed dataset (see Table S2).

### Analytical cohort

**Fig. 1:**
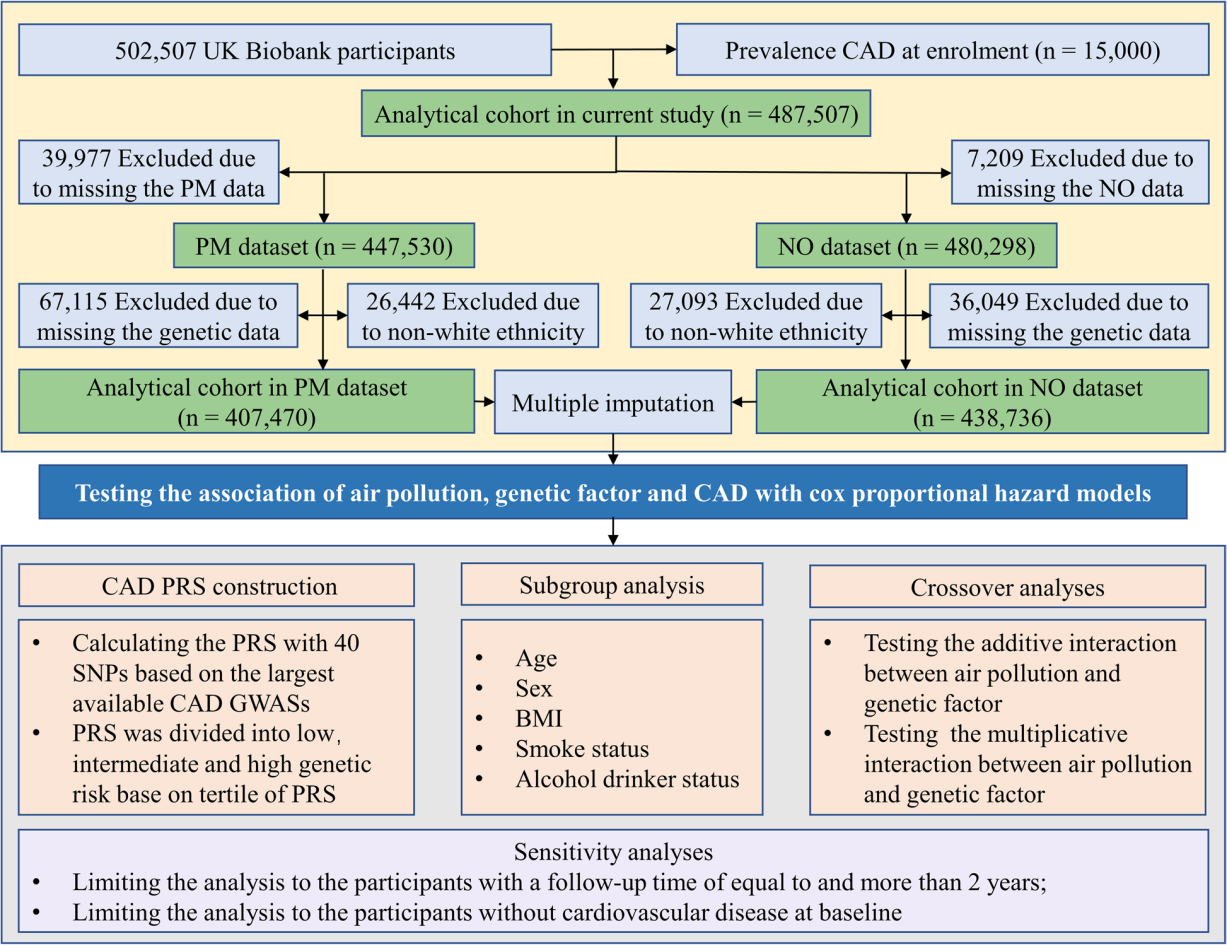
Flow diagram for participant inclusion. PM_2.5_, fine particulate matter with a diameter ≤ 2.5 μm; PM_10_, particulate matter with diameter ≤ 10 μm; PM_2.5−10_, particulate matter with diameter between 2.5 and 10 μm; NO_2_, nitrogen dioxide; NOx, nitrogen oxides. CAD, coronary artery disease; PRS, polygenic risk score; GWAS, genome-wide association study

Participants were excluded if they met one of the following criteria: (i) preexisting CAD at enrolment, (ii) lack of air pollution exposure information, or (iii) lack of CAD genetic information (Fig. 1). After exclusion for the above reasons, 447,530 subjects were used to investigate the association of particulate matter (PM_2.5_, PM_2.5−10_, PM_10_) and CAD, and 480,298 subjects were used to investigate the association of nitrogen oxides (NO_2_, NO_x_) and CAD. Furthermore, when considering the genetic factors, there were 407,470 individuals to investigate the relationship of PM, genetic factors and CAD and 438,736 in the NO group.

### Statistical analyses

All analyses in the current study were conducted using R software (Version 4.1.1) and Stata (Version 15.1), and two-sided *P* values < 0.05 were defined as statistically significant. Cox regression models were constructed to investigate the relationship between air pollution and CAD and to calculate hazard ratios (HRs) and 95% confidence intervals (CIs) with adjustment for multiple covariates. We tested the proportional hazards assumption with Schoenfeld residuals methods. We performed a restricted cubic spline (RCS) transformation to explore possible linear/nonlinear correlations of air pollution and CAD and used the Akaike information criterion (AIC) to determine the optimal number of knots of RSC transformation [27].

Because more than 90% of participants are white and race is a common confounding factor, we limited subjects to the White race to explore the combined effects of air pollution exposure and genetic factors on CAD incidence. The additive interaction term was assessed with two indices: the relative excess risk due to the interaction (RERI) and the attributable proportion (AP) due to the interaction [28]. The 95% CIs of the RERI and AP were determined using Excel written by T. Andersson [29], and when 0 was within the CIs of the RERI and AP, it meant that there was no additive interaction. The multiplicative interaction term was assessed by setting variable cross-product terms of gene‒environmental factors in the models to observe whether the corresponding *P* value was < 0.05.

Multiple imputation was conducted to account for the missing covariate data. The missing categorical variables were imputed with multiple imputation based on latent class (MILC), and the missing continuous variables (e.g., physical activity) were imputed with multivariate imputation by chained equation (MICE) using predictive mean matching. To validate the robustness of our results, we perform several sensitivity analyses. Considering the characteristics of the cohort study, we excluded participants with less than 2 years of follow-up time to avoid false-positive associations.

## Results

Table 1 presents the baseline characteristics of eligible participants by incident CAD. In the PM dataset, participants who suffered from CAD were mainly males, older, and had a higher BMI than controls (all *P* < 0.001). Moreover, previous or current smokers were more likely to develop CAD, while the opposite is true for current alcohol drinkers (all *P* < 0.001). Additionally, participants with diabetes or CVD at baseline were more likely to develop CAD (all *P* < 0.001). Participants in the NOx dataset had similar descriptions of baseline characteristics (all *P* < 0.001). In addition, PM_2.5_ was highly correlated with nitrogen oxides (*r* = 0.86 for NO_2_; *r* = 0.85 for NO_x_), while PM_2.5−10_ was related to PM_10_ (*r* = 0.82) (see Figure S1).

**Table 1:** Baseline characteristics of participants in the UK Biobank study

Characteristic	Incident coronary artery disease					
	PM dataset (*n* = 447,530)			NO dataset (*n* = 480,298)		
	Yes (*n* = 22,897)	No (*n* = 464,610)	*P*	Yes (*n* = 22,897)	No (*n* = 464,610)	*P*
Age (years, mean ± SD)	61.0 ± 6.5	56.2 ± 8.1	< 0.001	61.0 ± 6.5	56.1 ± 8.1	< 0.001
Male (%)	14,122 (66.7)	186,112 (43.7)	< 0.001	15,109 (66.8)	199,348 (43.6)	< 0.001
White (%)	19,741 (93.4)	399,740 (93.9)	< 0.001	21,118 (93.6)	430,436 (94.2)	< 0.001
BMI (kg/m^2^, mean ± SD)	29.0 ± 5.0	27.3 ± 4.8	< 0.001	29.0 ± 5.0	27.3 ± 4.8	< 0.001
BMI (kg/m^2^, %)			< 0.001			< 0.001
Normal (< 25 kg/m^2^)	4,357 (20.6)	144,450 (33.9)		4,607 (20.4)	155,268 (33.9)	
Overweight (25 to 29.9 kg/m^2^)	9,183 (43.3)	179,600 (42.1)		9,852 (43.6)	192,827 (42.1)	
Obesity (≥ 30 kg/m^2^)	7,443 (35.1)	99,791 (23.4)		7,924 (35.0)	106,987 (23.4)	
Missing value	206 (1.0)	2,500 (0.6)		225 (1.0)	2,608 (0.6)	
Physical activity (MET, min/week, mean ± SD)	2634.0 ± 2503.0	2664.8 ± 2431.7	0.072	2625.2 ± 2497.3	2656.4 ± 2424.2	0.059
Education level			< 0.001			< 0.001
College or University degree	4,758 (22.8)	137,556 (32.6)		5,149 (23.1)	150,059 (33.1)	
A/AS-level	1,732 (8.3)	47,642 (11.3)		1,863 (8.4)	51,397 (11.3)	
O-level/GCSE	4,084 (19.5)	92,011 (21.8)		4,263 (19.1)	97,050 (21.4)	
CSE	878 (4.2)	24,807 (5.9)		894 (4.0)	25,195 (5.6)	
NVQ/HND/HNC	1,869 (8.9)	27,036 (6.4)		1,969 (8.8)	29,005 (6.4)	
Other qualifications (nurse)	1,220 (5.9)	21,521 (5.1)		1,302 (5.8)	23,283 (5.1)	
None	6,359 (30.4)	71,643 (16.9)		6,873 (30.8)	77,548 (17.1)	
TDI (mean ± SD)	-0.97 ± 3.2	-1.4 ± 3.0	< 0.001	-1.8 ± 3.3	-1.4 ± 3.1	< 0.001
Smoke status (%)			< 0.001			< 0.001
Never	8,844 (41.8)	237,242 (55.7)		9,445 (41.8)	255,109 (55.7)	
Previous	9,028 (42.6)	143,604 (33.7)		9,567 (42.3)	153,341 (33.5)	
Current	3,138 (14.8)	43,090 (10.1)		3,403 (15.1)	46,712 (10.2)	
Missing value	179 (0.8)	2,405 (0.5)		193 (0.8)	2,528 (0.6)	
Alcohol drinker status (%)			< 0.001			< 0.001
Never	1,201 (5.7)	18,636 (4.4)		1,273 (5.6)	19,921 (4.4)	
Previous	1,209 (5.7)	14,429 (3.4)		1,302 (5.8)	15,522 (3.4)	
Current	18,686 (88.2)	391,901 (91.9)		19,933 (88.2)	420,804 (91.9)	
Missing value	93 (0.4)	1,375 (0.3)		100 (0.4)	1,443 (0.3)	
Diabetes baseline (%)	3,013 (14.2)	18,858 (4.4)	< 0.001	3,176 (14.1)	20,078 (4.4)	< 0.001
CVD baseline (%)	5,210 (24.7)	9,850 (2.3)	< 0.001	5,573 (24.7)	10,728 (2.4)	< 0.001

Data were presented as mean ± SD, numbers and (percentages). The comparison of continuous variables was performed with t-test, and categorical variables was performed with χ-test

*SD* standard deviation, *MET* Metabolic Equivalent Task, *BMI* body mass index, *TDI* Townsend Deprivation index

During a median of 8.8 years (4,187,431 person-years) of follow-up, 22,897 incident CAD cases were recorded. Kaplan‒Meier (K‒M) plots for CAD in the PM_2.5_, PM_10_, PM_2.5−10_, NO_2_ and NO_x_, concentration quartiles are presented in Figures S2-S6, respectively. The results showed that the differences in Kaplan‒Meier curves were significant among PM_2.5_ (*P* = 4.0 × 10^−13^), PM_10_ (*P* = 0.010), NO_2_ (*P* = 9.0 × 10^−11^) and NO_x_ (*P* = 6.0 × 10^−14^) concentration quartiles but not PM_2.5−10_ (*P* = 0.200).

Table 2 presents the relationships between air pollution exposure and CAD risk after adjustment for multiple possible confounders. The results showed that PM_2.5_, PM_10_, NO_2_ and NO_x_ each was still related to an elevated risk of CAD in the multivariate-adjusted models (all *P* < 0.05). In the model 1, the HRs (95% CI) of CAD for 10-µg/m^3^ increases in PM_2.5_, PM_10_, NO_2_ and NO_x_ concentrations were 2.56 (2.27, 2.90), 1.22 (1.14, 1.31), 1.11 (1.09, 1.13) and 1.05 (1.04, 1.06), respectively. After further adjusting for race, drinking, smoking status, education level, BMI, UK Biobank assessment centre, physical activity, diabetes and CVD at baseline, the HRs (95% CI) of CAD for 10-µg/m^3^ increases in PM_2.5_, NO_2_ and NO_x_ concentrations were 1.25 (1.09, 1.44), 1.03 (1.01, 1.05) and 1.01 (1.00, 1.02), respectively. The relationship between PM_2.5−10_ and PM_10_ exposure and CAD risk were not statistically significant (*P* = 0.865 for PM_2.5−10_; *P* = 0.146 for PM_10_). Certainly, the third quartiles of PM_10_ concentration presented more significant effect on CAD risk than first quartiles (HRs [95% CI]: 1.05 [1.01, 1.09], *P* = 0.027). Interestingly, the association between air pollutants and CAD were not statistically significant after further adjusting TDI (All *P* > 0.05, Table S3). Furthermore, the RCS results also verified the significant association of air pollutants, including PM_2.5_ (*P* < 0.0001), PM_10_ (*P* = 0.0001), NO_2_ (*P* < 0.0001) and NO_x_ (*P* < 0.0001), and CAD risk (Figures S7). In addition, subgroup analysis showed that PM_2.5_, NO_2_ and NO_x_ had more significant effects on CAD risk in female group, overweight/obesity group and previous or current smokers (see Table S4). And NO_x_ and sex have an interaction effect on CAD risk.

**Table 2:** Adjusted hazard ratio and 95% confidence interval of coronary artery disease by air pollution exposure

Air pollutants		Air pollution concentration quartiles				Per 10 μg/m^3^ increment	*P*
		First	Second	Third	Fourth		
PM_2.5_	Concentration (μg/m^3^, range)	8.2–9.3	9.4–9.9	10.0–10.6	10.7–21.3		
	No. of cases	4,916	5,258	5,434	5,581		
	Model 1	1	1.12 (1.08, 1.16)	1.18 (1.14, 1.23)	1.30 (1.25, 1.35)	2.56 (2.27, 2.90)	< 2.00E-16
	Model 2	1	1.05 (1.01, 1.09)	1.05 (1.01, 1.09)	1.06 (1.02, 1.10)	**1.25 (1.09, 1.44)**	**0.001**
PM_2.5–10_	Concentration (μg/m^3^, range)	5.6–5.8	5.9–6.1	6.2–6.6	6.7–12.8		
	No. of cases	5,276	5,387	5,224	5,302		
	Model 1	1	1.05 (1.01, 1.09)	1.06 (1.02, 1.10)	1.07 (1.03, 1.11)	1.16 (1.00, 1.35)	0.045
	Model 2	1	1.01 (0.97, 1.05)	1.02 (0.98, 1.06)	1.02 (0.98, 1.06)	1.01 (0.87, 1.18)	0.865
PM_10_	Concentration (μg/m^3^, range)	11.8–15.3	15.4–16.0	16.1–17.0	17.1–31.4		
	No. of cases	5,154	5,415	5,371	5,249		
	Model 1	1	1.07 (1.03, 1.11)	1.12 (1.07, 1.16)	1.09 (1.05, 1.13)	1.22 (1.14, 1.31)	1.56E-08
	Model 2	1	1.02 (0.98, 1.06)	1.05 (1.01, 1.09)	1.02 (0.98, 1.06)	**1.06 (0.98, 1.14)**	**0.146**
NO_2_	Concentration (μg/m^3^, range)	12.9–21.5	21.6–26.2	26.3–31.3	31.4–108.5		
	No. of cases	5,267	5,720	5,950	5,671		
	Model 1	1	1.12 (1.08, 1.16)	1.20 (1.16, 1.25)	1.24 (1.20, 1.29)	1.11 (1.09, 1.13)	< 2.00E-16
	Model 2	1	1.02 (0.98, 1.06)	1.07 (1.03, 1.11)	1.06 (1.01, 1.10)	**1.03 (1.01, 1.05)**	**0.002**
NO_x_	Concentration (μg/m^3^, range)	19.7–34.4	34.5–42.4	42.5–50.8	50.9–265.9		
	No. of cases	5,175	5,702	5,855	5,876		
	Model 1	1	1.13 (1.09, 1.18)	1.22 (1.17, 1.27)	1.29 (1.25, 1.34)	1.05 (1.04, 1.06)	< 2.00E-16
	Model 2	1	1.04 (1.00, 1.08)	1.08 (1.04, 1.12)	1.06 (1.02, 1.11)	**1.01 (1.00, 1.02)**	**0.004**

Model 1, Cox proportional hazard model, adjusted for age (continuous), sex (male/female)

Model 2, model 1 plus race (White/Mixed /Asian or Asian British/Black or Black British), alcohol consumption (never/previous/current/missing), smoking status (never/previous/current/missing), body mass index (< 25 kg/m^2^/25 to 29.9 kg/m^2^/ ≥ 30 kg/m^2^/ missing), education level (College or University degree, A/AS-level, O-level/GCSE, CSE, NVQ/HND/HNC, other qualifications, none), UK Biobank assessment center, physical activity (continuous, MET-min/week), diabetes at baseline (yes/no), and cardiovascular disease at baseline (yes/no)

*PM*_*2.5*_ fine particulate matter with diameter ≤ 2.5 μm, *PM*_*2.5–10*_ particulate matter with diameter between 2.5 μm and 10 μm, *PM*_*10*_ particulate matter with diameter ≤ 10 μm, *NO*_*2*_ nitrogen dioxide, *NO*_*x*_ nitrogen oxides

**Fig. 2:**
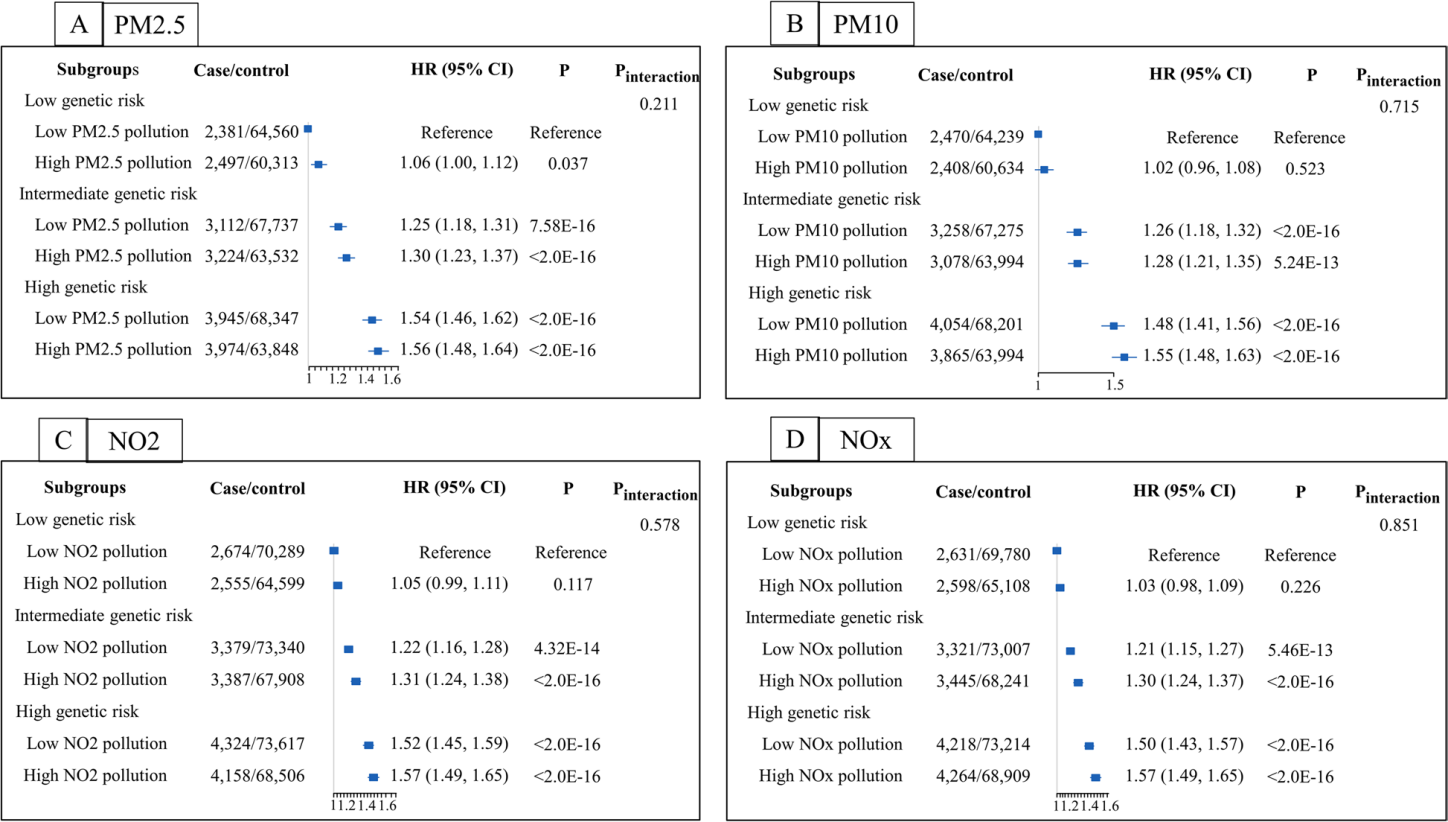
The joint association of the included air pollutant exposure and genetic categories with the risk of incident CAD in the UK Biobank. **A** Joint effects of PM_2.5_ and genetic variations; (**B**) Joint effects of PM_10_ and genetic variations; (**C**) Joint effects of NO_2_ and genetic variations; (**D**) Joint effects of NO_x_ and genetic variations. Adjusted for age (continuous), sex (male/female), alcohol consumption (never, previous, current, missing), smoking status (never, previous, current, missing), body mass index (< 25 kg/m^2^, 25 to 29.9 kg/m^2^, ≥ 30 kg/m^2^, missing), education level (College or University degree, A/AS-level, O-level/GCSE, CSE, NVQ/HND/HNC, other qualifications, none), UK Biobank assessment center, physical activity (continuous, MET-min/week), diabetes at baseline (yes/no), cardiovascular disease at baseline (yes/no), genotyping batch, and the first 4 genetic principal components

Then, we explored the combined effects of genetic factors and air pollution on the risk of CAD and found the statistically significant results (see Fig. 2). Although the multiplicative interactions of the genetic variation and air pollution on CAD risk were not statistically significant (all *P-interaction* > 0.05), we observed that individuals with high PRS and air pollution exposure simultaneously showed the highest risk of CAD compared with any other group. The HRs (95% CI) of CAD in the PM_2.5_, PM_10_, NO_2_ and NO_x_ high joint exposure groups were 1.56 (1.48, 1.64), 1.55 (1.48, 1.63), 1.57 (1.49, 1.65), and 1.57 (1.49, 1.65), respectively.

In addition, the RERI and AP were statistically significant, which demonstrated the positive additive interactions of genetic factors and air pollutants on CAD risk (see Table 3). Compared with the low genetic risk and low air pollution exposure group, the RERIs (95% CI) of CAD in the PM_2.5_, PM_10_, NO_2_ and NO_x_ high joint exposure groups were 0.12 (0.05, 0.19), 0.17(0.10, 0.24), 0.14 (0.07, 0.21), and 0.17 (0.10, 0.24), respectively, and the APs (95% CI) of CAD in the PM_2.5_, PM_10_, NO_2_ and NO_x_ high joint exposure groups were 0.09 (0.04, 0.14), 0.12(0.07, 0.18), 0.11 (0.06, 0.16), and 0.13 (0.08, 0.18), respectively. Specifically, in the high PM_2.5_ exposure and high PRS group, participants had an additional 13% risk of CAD compared with those with low PM_2.5_ exposure and a low PRS due to the synergistic effect of PM_2.5_ exposure and genetic risk; the gene-PM_2.5_ interaction was responsible for 10% of the CAD cases in the participants with high PM_2.5_ exposure and high genetic risk.

**Table 3:** Additive joint interaction for included air pollutants exposure and genetic categories on the incident coronary artery disease

Air pollution^b^	CAD PRS (tertiles)^c^			
	Intermediate^a^		High^a^	
	RERI (95% CI)	AP (95% CI)	RERI (95% CI)	AP (95% CI)
PM_2.5_				
High pollution	0.26 (0.20, 0.32)	0.28 (0.22, 0.33)	0.12 (0.05, 0.19)	0.09 (0.04, 0.14)
PM_10_				
High pollution	0.23 (0.17, 0.29)	0.25 (0.19, 0.31)	0.17 (0.10, 0.24)	0.12 (0.07, 0.18)
NO_2_				
High pollution	0.29 (0.24, 0.35)	0.31 (0.26, 0.36)	0.14 (0.07, 0.21)	0.11 (0.06, 0.16)
NO_x_				
High pollution	0.30 (0.25, 0.36)	0.32 (0.27, 0.37)	0.17 (0.10, 0.24)	0.13 (0.08, 0.18)

Adjusted for age (continuous), sex (male/female), alcohol consumption (never, previous, current, missing), smoking status (never, previous, current, missing), body mass index (< 25 kg/m^2^, 25 to 29.9 kg/m^2^, ≥ 30 kg/m^2^, missing), education level (College or University degree, A/AS-level, O-level/GCSE, CSE, NVQ/HND/HNC, other qualifications, none), UK Biobank assessment center, physical activity (continuous, MET-min/week), diabetes at baseline (yes/no), cardiovascular disease at baseline (yes/no), genotyping batch, and the first 4 genetic principal components

*RERI* relative excess risk due to interaction, *AP* attributable proportion due to interaction, *CI* confidence interval, *PRS* polygenic risk score, *CAD* coronary artery disease, *PM*_*2.5*_, fine particulate matter with diameter ≤ 2.5 μm, *PM*_*10*_ particulate matter with diameter ≤ 10 μm, *NO*_*x*_ nitrogen oxides, *NO*_*2*_ nitrogen dioxide

^a^Defined by polygenic risk score: low (lowest tertiles), intermediate (second tertiles) and high (highest tertiles)

^b^Defined by median of air pollutants including PM_2.5_, PM_10_, NO_2_, and NO_x_ concentration

^c^To estimate RERI and AP, the lower air pollution category and the lowest genetic risk (low PRS) groups were the reference categories

Sensitivity analyses showed that the additive interactions and combined effects of genetic factors and air pollution remained statistically significant after excluding the individuals with a follow-up time of less than two years (see Tables S5, Tables S6-S9).

## Discussion

In this prospective cohort study with a median of 8.8 years of follow-up, we observed that air pollution exposure was significantly related to an increased CAD risk, and the risk could be further strengthened by CAD gene susceptibility. Additionally, there were positive gene‒environmental additive interactions on the onset of CAD.

Air pollutants are complex mixtures containing various different gases, liquids and particulates, whose complexities and biological responses make it difficult to demonstrate the relationship between different pollutants and diseases [30, 31]. Previous population-based studies have reported that PM_2.5_ exposure was relevant to both cardiovascular diseases and all-cause mortality [32, 33], and our results further verified this finding. Moreover, we found that PM_10_ exposure was also linked to an increased risk of CAD, but the association of PM_2.5−10_ and CAD risk was not statistically significant. The possible reason was that the effect of PM_10_ might rely on PM_2.5_ and PM10 comprising PM_2.5−10_ and PM_2.5_ [34]. To our knowledge, PM_2.5_ has the lowest particulate matter compared to PM_2.5−10_ and PM_10_, subsequently having the strongest potential toxicity, which is also responsible for an extensive proportion of the effects of PM_10_ [31]. Moreover, we also found significantly positive associations between NO_2_ and NO_x_ and CAD risk, even though the effects were lower than those of PM. Nitrogen oxides, other components of air pollutant mixtures, were also reported to be significantly related to the risk of CVD, but the results remained inconsistent due to short-term air pollution exposure [35, 36]. In addition, it is well known that older age and male sex are risk factors for CAD [1]. Our results further verified these findings. We also found that PM_2.5_, NO_2_ and NO_x_ had interactions with sex on CAD risk. NO_x_ and age have an interaction effect on CAD risk.

Previous animal studies have shown that particulate components may promote the growth of atherosclerotic plaques and add markers of plaque rupture vulnerability [37, 38]. Subsequently, some potential mechanisms linking air pollution and CVD have been demonstrated. Of those mechanisms, oxidative stress and inflammation are the most acceptable mechanisms accounting for the observed associations of air pollution and CAD [31, 39, 40]. Prolonged or repeated oxidative stress and inflammation may cause endothelial dysfunction, asymptomatic atherosclerosis, coronary vasomotor abnormalities, coronary artery calcium, acute myocardial infarction and coronary plaques, which could eventually develop into CAD [41–43].

In addition to environmental effects, genetic factors are also an important influencing factor of CAD risk [1, 44]. Previous studies and genome-wide association studies (GWAS) have demonstrated that CVD has a strong genetic component, with heritability estimates ranging between 40 and 60% [26, 45, 46]. Of course, the genetic effects are composed of multiple common single genomes. Indeed, GWAS have shown that most cardio-metabolic diseases, including diabetes, hypertension and CAD, are influenced by many risk alleles [47]. In this study, we found that participants with high genetic risk and high air pollution levels were prone to developing CAD compared to subjects with low genetic risk and low air pollution based on the PRS. Furthermore, air pollution and genetic factors presented clear additive interactions on CAD risk. To the best of our knowledge, this is the first large-scale prospective study to evaluate the combined and interaction effects of air pollution and genetic factors on the incidence of CAD. Until now, limited previous studies only assessed the combination and interactions of the gene‒environment in some common CVDs, such as hypertension [16, 18, 19]. Certainly, a large study [48] with approximately 60 919 CAD cases and 80 243 controls revealed that the protective effect of *ADAMTS7* gene polymorphism (rs7178051) on CAD was weakened by environmental factors (5% lower CAD risk in smokers; 12% lower CAD risk in nonsmokers) compared to nonsmokers (12% lower risk), which showed the gene‒environmental interaction effects on CAD risk from another aspect. Considering the reverse causality and confounding bias, when excluding the participants with a follow-up time of less than two years or with baseline CVD, we repeated the analysis procedure, and the results did not change appreciably.

There are some strengths in our study. Our study was the first prospective comprehensive study to evaluate the separate, combined and interaction effects of air pollution and genetic factors on the risk of CAD. This new viewpoint provides clues to the aetiology of CAD and provides a reference for the prevention and treatment of susceptible people. Moreover, our article has strict quality control, including strict assessment of air pollution exposure and outcomes, proper statistical methods (PRS), further guaranteeing the robustness and reliability of our findings.

However, we must acknowledge that our study also has some shortcomings. First, air pollutants are made up of many different components [30, 31], but we only explored the associations between some common pollutants, including PM_2.5_, PM_2.5−10_, PM_10_, NO_2_ and NO_x_ and CAD risk, ignoring the specific parts of air pollutant chemical constituents. Further studies may concentrate on the associations of certain components or physical, chemical or biological properties of air pollutant components and CAD risk [49, 50]. Second, air pollution exposure may be misclassified because the exposure data for this study were only restricted to residential addresses. Although the coverage area of the monitoring system is up to 400 km, the specific real-time environmental exposure of the participants cannot be detected. Future research should consider adorning high-tech portable devices to detect the participants’ surrounding environment in real time. Third, the data were obtained from UK populations, most of which were white (94.0%). Therefore, the generalization of gene-associated findings should be interpreted with caution. Meanwhile, in the future, studies on the respective and combined effects of air pollution-genetic factors on CAD could be conducted in different ethnic groups. Fourth, air pollutant levels were a time-varying factor, but the air pollution information in the UK Biobank was available for several years (2010 for PM_2.5_, PM_2.5−10_, and NO_x_; 2005–2007 and 2010 for NO_2_; 2007 and 2010 for PM_10_). If applicable, further study should dynamically detect the air pollutant concentration to avoid over- or underestimating air pollutant toxicity. Fifth, although education level and the Townsend deprivation index are possibly associated with CAD, we did not include them as covariables because they were not available for us in the UK Biobank dataset. However, we included as many variables as possible that were associated with CAD, such as common metabolic disease, physical activity, smoking and alcohol consumption status. Certainly, it is necessary to consider these influencing factors, including education level and the Townsend deprivation index, in future studies related to CAD.

## Conclusions

In summary, air pollution exposure was significantly associated with an increased CAD risk, which could be further strengthened by CAD. Additionally, our findings showed positive additive gene‒environmental interactions on the onset of CAD and highlighted the importance of comprehensively evaluating air pollution and genetic factors in prevention efforts for people who are vulnerable to CAD.

### Supplementary Information

**Additional file 1:** **Figure S1. **Correlation heatmap of air pollutant exposures calculated using data from 447,530 samples. **Figure S2.** Kaplan‒Meier curve for the incidence of coronary artery disease in individuals exposed to PM_2.5_ with quartiles of concentrations. **Figure S3.** Kaplan‒Meier curve for the incidence of coronary artery disease in individuals exposed to PM_2.5-10_ with quartiles of concentrations. **Figure S4.** Kaplan‒Meier curve for the incidence of coronary artery disease in individuals exposed to PM_10_ with quartiles of concentrations. **Figure S5.** Kaplan‒Meier curve for the incidence of coronary artery disease in individuals exposed to NO_2_ at quartiles of concentrations. **Figure S6.** Kaplan‒Meier curve for the incidence of coronary artery disease in individuals exposed to NO_x_ at quartiles of concentrations. **Figure S7.** Association between air pollution concentration and the risk of CAD. **Table S1. **Code list of coronary artery disease in the UK Biobank. **Table S2. **The main information on genetic variants associated with coronary artery disease in the UK Biobank. **Table S3. **Adjusted hazard ratio and 95% confidence interval of coronary artery disease by air pollution exposure. **Table S4.** Subgroup analysis for the association of coronary artery disease per 10 μg/m^3^ increase in air pollutants by specific characteristics. **Table S5. **Additive joint interaction for included air pollutant exposure and genetic categories on incident coronary artery disease after excluding participants with a follow-up time of less than 2 years in the UK Biobank. **Table S6. **The joint association of PM_2.5_ exposure and coronary artery disease PRS with the risk of incident coronary artery disease after excluding participants with a follow-up time of less than 2 years in the UK Biobank. **Table S7.**The joint association of PM_10_ exposure and coronary artery disease PRS with the risk of incident coronary artery disease after excluding participants with a follow-up time of less than 2 years in the UK Biobank. **Table S8. **The joint association of NO_2_ exposure and coronary artery disease PRS with the risk of incident coronary artery disease after excluding participants with a follow-up time of less than 2 years in the UK Biobank. **Table S9. **The joint association of NO_x_ exposure and coronary artery disease PRS with the risk of incident coronary artery disease after excluding participants with a follow-up time of less than 2 years in the UK Biobank.

## Data Availability

The data that support the findings of this study are available from UK Biobank project site, subject to registration and application process. Further details can be found at https://www.ukbiobank.ac.uk.

## Abbreviations

AP: Attributable proportion due to the interaction
AIC: Akaike information criterion
CAD: Coronary artery disease
CHD: Coronary heart disease
CVD: Cardiovascular disease
CI: Confidence interval
HR: Hazard ratio
ICD: International Classification of Diseases
NRAP: Near-roadway air pollution
NO2: Nitrogen dioxide
NOx: Nitrogen oxides
PRS: Polygenic risk score
PM: Particulate matter
RERI: Relative excess risk due to the interaction
RCS: Restricted cubic spline
SNP: Single nucleotide polymorphisms
