# Supplementary material for: Association of air pollution exposure and increased coronary artery disease risk: the modifying effect of genetic susceptibility
Source: Environ Health. 2023 Dec 8;22:85. doi: 10.1186/s12940-023-01038-y (PMC10704645; doi:10.1186/s12940-023-01038-y)
Supplement: Supplementary file 1 — Additional file 1: Figure S1. Correlation heatmap of air pollutant exposures calculated using data from 447,530 samples. Figure S2. Kaplan‒Meier curve for the incidence of coronary artery disease in individuals exposed to PM2.5 with quartiles of concentrations. Figure S3. Kaplan‒Meier curve for the incidence of coronary artery disease in individuals exposed to PM2.5-10 with quartiles of concentrations. Figure S4. Kaplan‒Meier curve for the incidence of coronary artery disease in individuals exposed to PM10 with quartiles of concentrations. Figure S5. Kaplan‒Meier curve for the incidence of coronary artery disease in individuals exposed to NO2 at quartiles of concentrations. Figure S6. Kaplan‒Meier curve for the incidence of coronary artery disease in individuals exposed to NOx at quartiles of concentrations. Figure S7. Association between air pollution concentration and the risk of CAD. Table S1. Code list of coronary artery disease in the UK Biobank. Table S2. The main information on genetic variants associated with coronary artery disease in the UK Biobank. Table S3. Adjusted hazard ratio and 95% confidence interval of coronary artery disease by air pollution exposure. Table S4. Subgroup analysis for the association of coronary artery disease per 10 μg/m3 increase in air pollutants by specific characteristics. Table S5. Additive joint interaction for included air pollutant exposure and genetic categories on incident coronary artery disease after excluding participants with a follow-up time of less than 2 years in the UK Biobank. Table S6. The joint association of PM2.5 exposure and coronary artery disease PRS with the risk of incident coronary artery disease after excluding participants with a follow-up time of less than 2 years in the UK Biobank. Table S7.The joint association of PM10 exposure and coronary artery disease PRS with the risk of incident coronary artery disease after excluding participants with a follow-up time of less than 2 years in the UK Biobank. [file 12940_2023_1038_MOESM1_ESM.docx]

**Supplementary materials**

**Association of air pollution exposure and increased coronary artery disease risk: the modifying effect of genetic susceptibility**

Zuqiang Fu, PhD, Yuanyuan Ma, MPH, Changjie Yang, MPH, Qian Liu, PhD, Jingjia Liang, PhD, Zhenkun Weng, PhD, Wenxiang Li, PhD, Shijie Zhou, PhD, Xiu Chen, PhD, Jin Xu, PhD, Cheng Xu, PhD, Tao Huang, PhD, Yong Zhou, PhD, Aihua Gu, PhD

**Contents**

**Supplementary Methods**

**Supplementary Figures and Tables**

**Figure S1.** Correlation heatmap of air pollutant exposures calculated using data from 447,530 samples.

**Figure S2**. Kaplan‒Meier curve for the incidence of coronary artery disease in individuals exposed to PM_2.5_ with quartiles of concentrations.

**Figure S3**. Kaplan‒Meier curve for the incidence of coronary artery disease in individuals exposed to PM_2.5-10_ with quartiles of concentrations.

**Figure S4**. Kaplan‒Meier curve for the incidence of coronary artery disease in individuals exposed to PM_10_ with quartiles of concentrations.

**Figure S5**. Kaplan‒Meier curve for the incidence of coronary artery disease in individuals exposed to NO_2_ at quartiles of concentrations.

**Figure S6.** Kaplan‒Meier curve for the incidence of coronary artery disease in individuals exposed to NO_x_ at quartiles of concentrations.

**Figure S7.** Association between air pollution concentration and the risk of CAD.

**Table S1.** Code list of coronary artery disease in the UK Biobank.

**Table S2.** The main information on genetic variants associated with coronary artery disease in the UK Biobank.

**Table S3.** Adjusted hazard ratio and 95% confidence interval of coronary artery disease by air pollution exposure.

**Table S4.** Subgroup analysis for the association of coronary artery disease per 10 μg/m^3^ increase in air pollutants by specific characteristics.

**Table S5.** Additive joint interaction for included air pollutant exposure and genetic categories on incident coronary artery disease after excluding participants with a follow-up time of less than 2 years in the UK Biobank.

**Table S6.** The joint association of PM_2.5_ exposure and coronary artery disease PRS with the risk of incident coronary artery disease after excluding participants with a follow-up time of less than 2 years in the UK Biobank.

**Table S7.** The joint association of PM_10_ exposure and coronary artery disease PRS with the risk of incident coronary artery disease after excluding participants with a follow-up time of less than 2 years in the UK Biobank.

**Table S8.** The joint association of NO_2_ exposure and coronary artery disease PRS with the risk of incident coronary artery disease after excluding participants with a follow-up time of less than 2 years in the UK Biobank.

**Table S9.** The joint association of NO_x_ exposure and coronary artery disease PRS with the risk of incident coronary artery disease after excluding participants with a follow-up time of less than 2 years in the UK Biobank.

**Supplementary Methods**

**Air pollution exposure assessment**

The land use regression (LUR) model can assess spatial variations in annual average ambient air pollutant concentrations through a series of geospatial predictive variables generated from geographic information systems (GIS), such as topography, population traffic intensity, and land use. Models were developed using a supervised stepwise method that maximized the model-explained variance, with a priori specified signs of slopes (e.g., positive for traffic intensity). Then, the models were optimized locally with no attempt to force a common model to all study areas [1]. The information on air pollution exposure was coupled with the records from residential addresses provided at the baseline visit. Notably, the ESCAPE estimates for PM were only valid within 400 km of the monitoring area (Greater London); therefore, all addresses more than 400 km away from Greater London were not assigned PM concentrations, which led to 33,935 missing values (<https://biobank.ndph.ox.ac.uk/showcase/label.cgi?id=114>).

Exposure information regarding PM_2.5_, PM_2.5-10_, and NO_x_ was collected by the UK Biobank in 2010, while information regarding NO_2_ and PM_10_ was available for several years (2005-2007 and 2010 for NO_2_; 2007 and 2010 for PM_10_). Therefore, we used the averaged values of NO_2_ and PM_10_ for the analyses.

**Calculation of** **covariates**

Based on items from the short International Physical Activity Questionnaire (IPAQ), metabolic equivalent task (MET) minutes were adopted to assess physical activity. Smoking or alcohol consumption status was classified as never smokers or drinkers or as previous or current smokers or drinkers. In addition, BMI was generated from height and weight data, which were measured by trained nurses during the baseline assessment. BMI was classified as normal (<25 kg/m^2^), overweight (25-29.9 kg/m^2^) and obese (≥30 kg/m^2^) according to the WHO criteria. Systolic blood pressure (SBP) and diastolic blood pressure (DBP) were measured at baseline using standard procedures by trained nurses. All the aforementioned procedures were conducted by skilled professionals, and the mean values of two automated or manual measurements were used.

The following information was available online from relevant data fields in the data showcase on the UK Biobank website ([https://biobank.ndph.ox.ac.uk/showcase](https://biobank.ndph.ox.ac.uk/showcase/search.cgi)/): age (Data field 21003), sex (Data field 31), race (Data field 21000), alcohol consumption (Data field 1558), smoking status (Data field 20116), BMI (Data field 21001), educational level (Data field 6138), UK Biobank assessment center (Data field 54), physical activity (Data field 6164), diabetes (Data field 2443) and CVD (Data field 6150).

**PRS contraction**

A detailed description of the procedure for genotyping and imputation in the UK Biobank has been described previously [2]. The PRS was calculated using the equation: PRS=β_1_×SNP_1_ + β_2_×SNP_2_ + … +β_k_×SNP_k_ + … +β_n_×SNP_n,_ where n was the total number of SNP_s_, and β_k_ was the per-allele log odds ratio (OR) for CAD associated with SNP_k [3]_. In addition, each SNP was recoded as 0, 1, or 2 according to the number of risk alleles to construct the PRS and the effect size estimates of β_k_ were derived from a previous study [4]. The participants were categorized as having low (lowest tertile), intermediate (middle tertile) and high (highest tertile) genetic risk of CAD based on the distribution of the PRS among the noncases, which was indicated to be effective [5, 6].

**References**

1. Vienneau D, de Hoogh K, Bechle MJ, Beelen R, van Donkelaar A, Martin RV, Millet DB, Hoek G, Marshall JD: **Western European land use regression incorporating satellite- and ground-based measurements of NO2 and PM10**. *Environmental science & technology* 2013, **47**(23):13555-13564.

2. Bycroft C, Freeman C, Petkova D, Band G, Elliott LT, Sharp K, Motyer A, Vukcevic D, Delaneau O, O’Connell J *et al*: **Genome-wide genetic data on ~500,000 UK Biobank participants.** *bioRxiv* 2017.

3. Huang Y, Zhu M, Ji M, Fan J, Xie J, Wei X, Jiang X, Xu J, Chen L, Yin R *et al*: **Air Pollution, Genetic Factors, and the Risk of Lung Cancer: A Prospective Study in the UK Biobank**. *American journal of respiratory and critical care medicine* 2021, **204**(7):817-825.

4. Nikpay M, Goel A, Won HH, Hall LM, Willenborg C, Kanoni S, Saleheen D, Kyriakou T, Nelson CP, Hopewell JC *et al*: **A comprehensive 1,000 Genomes-based genome-wide association meta-analysis of coronary artery disease**. *Nature genetics* 2015, **47**(10):1121-1130.

5. Arthur RS, Wang T, Xue X, Kamensky V, Rohan TE: **Genetic Factors, Adherence to Healthy Lifestyle Behavior, and Risk of Invasive Breast Cancer Among Women in the UK Biobank**. *Journal of the National Cancer Institute* 2020, **112**(9):893-901.

6. Wang M, Zhou T, Song Y, Li X, Ma H, Hu Y, Heianza Y, Qi L: **Joint exposure to various ambient air pollutants and incident heart failure: a prospective analysis in UK Biobank**. *European heart journal* 2021, **42**(16):1582-1591.

**
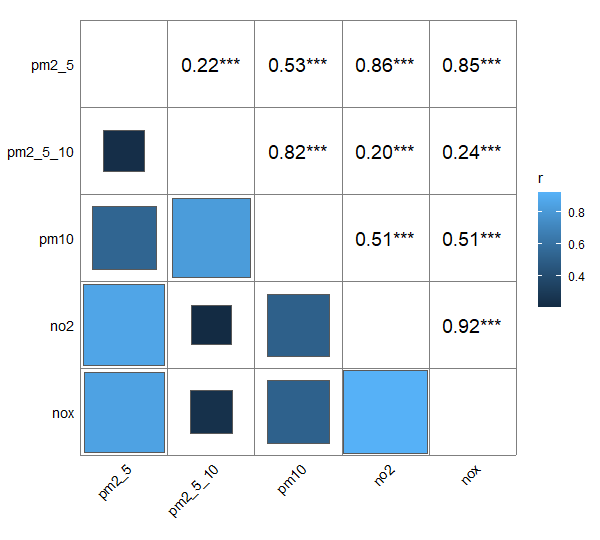
 Figure S1.** Correlation heatmap of air pollutant exposures calculated using data from 447,530 samples.

**
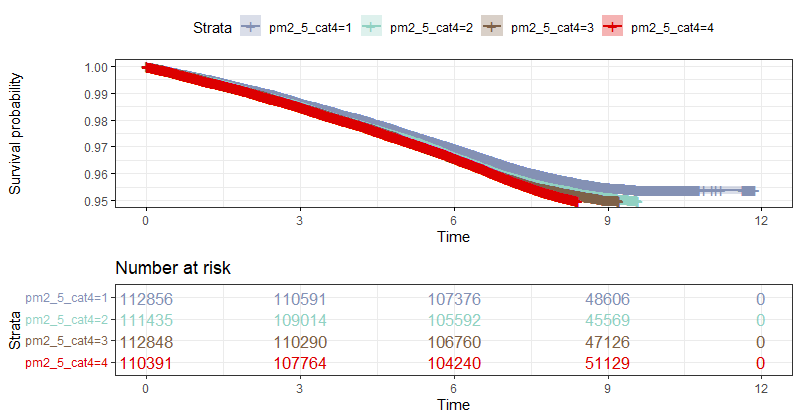
**

**Figure S2**. Kaplan‒Meier curve for the incidence of coronary artery disease in individuals exposed to PM_2.5_ with quartiles of concentrations.


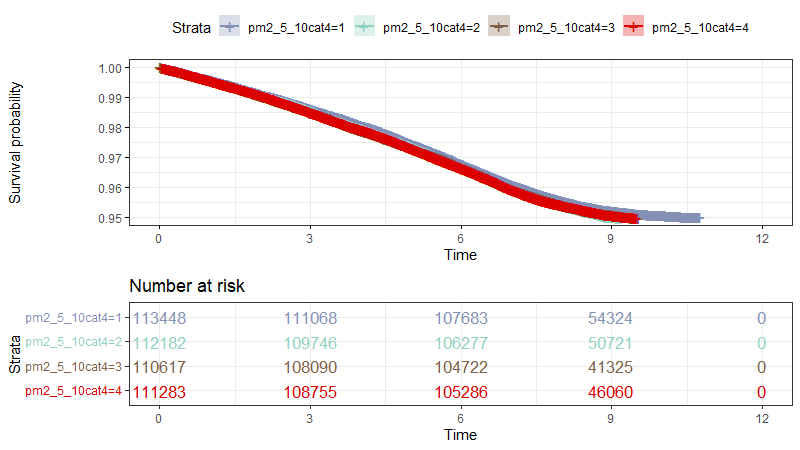


**Figure S3**. Kaplan‒Meier curve for the incidence of coronary artery disease in individuals exposed to PM_2.5-10_ with quartiles of concentrations.


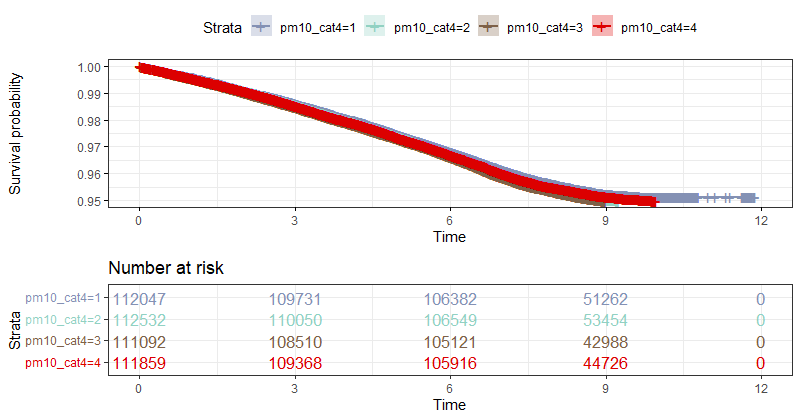


**Figure S4**. Kaplan‒Meier curve for the incidence of coronary artery disease in individuals exposed to PM_10_ with quartiles of concentrations.

**
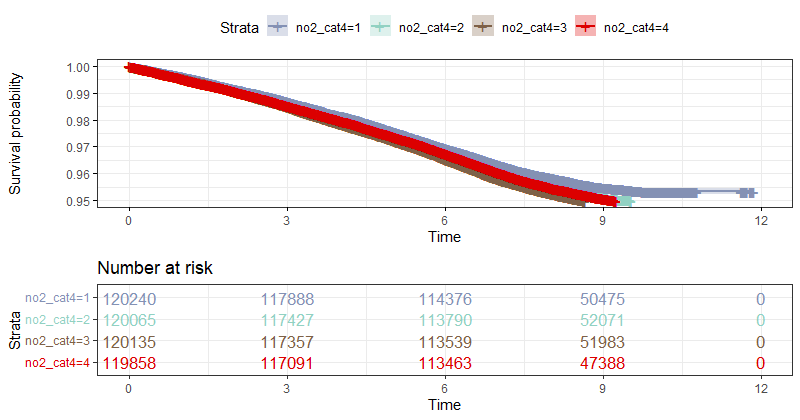
**

**Figure S5**. Kaplan‒Meier curve for the incidence of coronary artery disease in individuals exposed to NO_2_ at quartiles of concentrations.

**
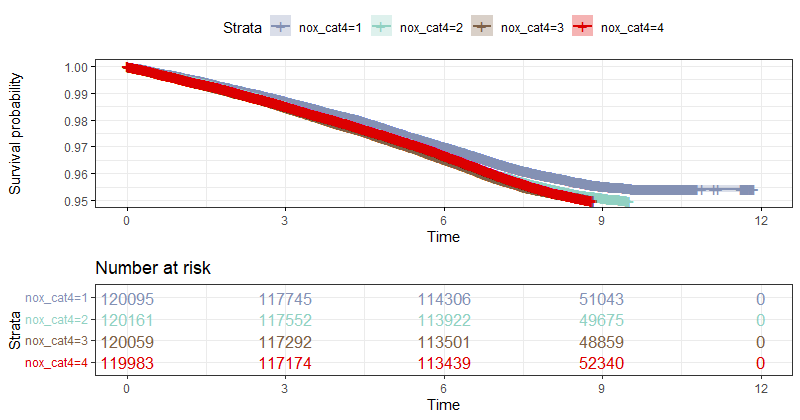
**

**Figure S6.** Kaplan‒Meier curve for the incidence of coronary artery disease in individuals exposed to NO_x_ at quartiles of concentrations.

**
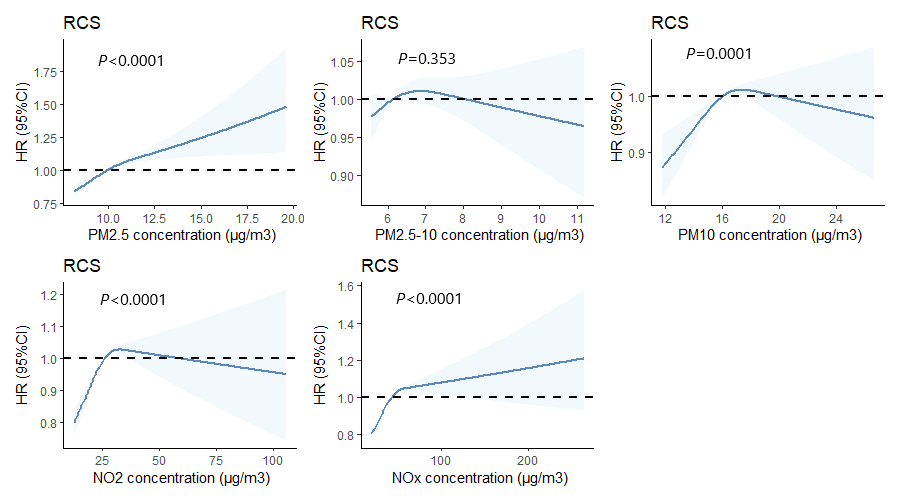
**

**Figure S7.** Association between air pollution concentration and the risk of CAD.

**Table S1.** Code list of coronary artery disease in the UK biobank.

| Hospital codes | | | Self-reported cases | | Diagnosed cases by doctor |
| --- | --- | --- | --- | --- | --- |
| ICD-10  (Biobank field: 41270) | ICD-9 (Biobank field: 41271) | OPCS-4 (Biobank field: 41272) | non-cancer illness code (Biobank field: 20002) | operation code (Biobank field: 20004) | Vascular/heart problems (Biobank field: 6150) |
| I21 | 410 | K40.1-4 | 1075: Heart attack/myocardial infarction | 1070: Coronary angioplasty | 1: Coronary angioplasty |
| I22 | 411 | K41.1-4 |  | 1095: Coronary artery bypass grafts |  |
| I23 | 412 | K45.1-5 |  |  |  |
| I24 |  | K49.1-2 |  |  |  |
| I25.2 |  | K49.8-9 |  |  |  |
|  |  | K50.2 |  |  |  |
|  |  | K75.1-4 |  |  |  |
|  |  | K75.8-9 |  |  |  |

ICD, International Classification of Diseases; OPCS, Office of Population Censuses and Surveys: Classification of interventions and Procedures.

**Table S2.** The main information on genetic variants associated with coronary artery disease in the UK Biobank.

| Chr | Rs ID | Lead effect/ non-effect allele | Effect (beta) | Lead effect allele frequency | *P* |
| --- | --- | --- | --- | --- | --- |
| 1 | rs7528419 | A/G | 0.113 | 0.786 | 1.97E-23 |
| 1 | rs9970807 | C/T | 0.122 | 0.915 | 5.00E-14 |
| 1 | rs67180937 | G/T | 0.077 | 0.663 | 1.01E-12 |
| 1 | rs6689306 | A/G | 0.058 | 0.448 | 2.60E-09 |
| 1 | rs11206510 | T/C | 0.077 | 0.848 | 2.34E-08 |
| 2 | rs7568458 | A/T | 0.058 | 0.449 | 3.62E-10 |
| 2 | rs17678683 | G/T | 0.095 | 0.088 | 3.00E-09 |
| 2 | rs16986953 | A/G | 0.086 | 0.105 | 1.45E-08 |
| 4 | rs4593108 | C/G | 0.068 | 0.795 | 8.82E-10 |
| 4 | rs72689147 | G/T | 0.068 | 0.817 | 6.07E-09 |
| 4 | rs17087335 | T/G | 0.058 | 0.210 | 4.60E-08 |
| 6 | rs9349379 | G/A | 0.131 | 0.432 | 1.81E-42 |
| 6 | rs55730499 | T/C | 0.315 | 0.056 | 5.39E-39 |
| 6 | rs4252185 | C/T | 0.293 | 0.060 | 1.64E-32 |
| 6 | rs12202017 | A/G | 0.068 | 0.700 | 1.98E-11 |
| 6 | rs56336142 | T/C | 0.068 | 0.807 | 1.85E-08 |
| 7 | rs11556924 | C/T | 0.077 | 0.687 | 5.34E-11 |
| 7 | rs2107595 | A/G | 0.077 | 0.200 | 8.05E-11 |
| 7 | rs3918226 | T/C | 0.131 | 0.060 | 1.7E-09 |
| 9 | rs2891168 | G/A | 0.191 | 0.489 | 2.29E-98 |
| 9 | rs2519093 | T/C | 0.077 | 0.191 | 1.19E-11 |
| 10 | rs1870634 | G/T | 0.077 | 0.637 | 5.55E-15 |
| 10 | rs1412444 | T/C | 0.068 | 0.369 | 5.15E-12 |
| 10 | rs2487928 | A/G | 0.058 | 0.418 | 4.41E-11 |
| 10 | rs11191416 | T/G | 0.077 | 0.873 | 4.65E-09 |
| 11 | rs2128739 | A/C | 0.068 | 0.324 | 7.05E-11 |
| 11 | rs10840293 | A/G | 0.058 | 0.550 | 1.30E-08 |
| 12 | rs2681472 | G/A | 0.077 | 0.201 | 6.17E-11 |
| 12 | rs3184504 | T/C | 0.068 | 0.422 | 1.03E-09 |
| 13 | rs11838776 | A/G | 0.068 | 0.263 | 1.83E-10 |
| 14 | rs10139550 | G/C | 0.058 | 0.423 | 1.38E-08 |
| 15 | rs4468572 | C/T | 0.077 | 0.586 | 4.44E-16 |
| 15 | rs56062135 | C/T | 0.068 | 0.790 | 4.5E-09 |
| 15 | rs8042271 | G/A | 0.095 | 0.900 | 3.70E-08 |
| 17 | rs7212798 | C/T | 0.077 | 0.150 | 1.90E-08 |
| 18 | rs663129 | A/G | 0.058 | 0.260 | 3.20E-08 |
| 19 | rs56289821 | G/A | 0.131 | 0.900 | 4.44E-15 |
| 19 | rs4420638 | G/A | 0.095 | 0.166 | 7.07E-11 |
| 21 | rs28451064 | A/G | 0.131 | 0.121 | 1.33E-15 |
| 22 | rs180803 | G/T | 0.182 | 0.970 | 1.6E-10 |

Chr, chromosome.

**Table S3.** Adjusted hazard ratio and 95% confidence interval of coronary artery disease by air pollution exposure.

| Air pollutants |  | Air pollution concentration quartiles | | | | Per 10 μg/m^3^ increment | *P* |
| --- | --- | --- | --- | --- | --- | --- | --- |
|  |  | First | Second | Third | Fourth |  |  |
| PM_2.5_ | Concentration (μg/m^3^, range) | 8.2-9.3 | 9.4-9.9 | 10.0-10.6 | 10.7-21.3 |  |  |
|  | No. of cases | 4,916 | 5,258 | 5,434 | 5,581 |  |  |
|  | Model 1 | 1 | 1.12 (1.08, 1.16) | 1.18 (1.14, 1.23) | 1.30 (1.25, 1.35) | 2.56 (2.27, 2.90) | <2.00E-16 |
|  | Model 2 | 1 | 1.04 (1.00, 1.08) | 1.02 (0.98, 1.07) | 1.00 (0.96, 1.05) | 1.02 (0.88, 1.18) | 0.780 |
| PM_2.5-10_ | Concentration (μg/m^3^, range) | 5.6-5.8 | 5.9-6.1 | 6.2-6.6 | 6.7-12.8 |  |  |
|  | No. of cases | 5,276 | 5,387 | 5,224 | 5,302 |  |  |
|  | Model 1 | 1 | 1.05 (1.01, 1.09) | 1.06 (1.02, 1.10) | 1.07 (1.03, 1.11) | 1.16 (1.00, 1.35) | 0.045 |
|  | Model 2 | 1 | 1.00 (0.97, 1.04) | 1.00 (0.96, 1.04) | 1.00 (0.96, 1.04) | 0.98 (0.84, 1.15) | 0.828 |
| PM_10_ | Concentration (μg/m^3^, range) | 11.8-15.3 | 15.4-16.0 | 16.1-17.0 | 17.1-31.4 |  |  |
|  | No. of cases | 5,154 | 5,415 | 5,371 | 5,249 |  |  |
|  | Model 1 | 1 | 1.07 (1.03, 1.11) | 1.12 (1.07, 1.16) | 1.09 (1.05, 1.13) | 1.22 (1.14, 1.31) | 1.56E-08 |
|  | Model 2 | 1 | 1.01 (0.97, 1.05) | 1.02 (0.98, 1.06) | 0.99 (0.95, 1.03) | 1.02 (0.94, 1.10) | 0.674 |
| NO_2_ | Concentration (μg/m^3^, range) | 12.9-21.5 | 21.6-26.2 | 26.3-31.3 | 31.4-108.5 |  |  |
|  | No. of cases | 5,267 | 5,720 | 5,950 | 5,671 |  |  |
|  | Model 1 | 1 | 1.12 (1.08, 1.16) | 1.20 (1.16, 1.25) | 1.24 (1.20, 1.29) | 1.11 (1.09, 1.13) | <2.00E-16 |
|  | Model 2 | 1 | 1.01 (0.97, 1.05) | 1.04 (1.00, 1.08) | 0.99 (0.95, 1.04) | 1.00 (0.98, 1.02) | 0.876 |
| NO_x_ | Concentration (μg/m^3^, range) | 19.7-34.4 | 34.5-42.4 | 42.5-50.8 | 50.9-265.9 |  |  |
|  | No. of cases | 5,175 | 5,702 | 5,855 | 5,876 |  |  |
|  | Model 1 | 1 | 1.13 (1.09, 1.18) | 1.22 (1.17, 1.27) | 1.29 (1.25, 1.34) | 1.05 (1.04, 1.06) | <2.00E-16 |
|  | Model 2 | 1 | 1.03 (0.99, 1.07) | 1.05 (1.01, 1.10) | 1.01 (0.97, 1.05) | 1.00 (0.99, 1.01) | 0.767 |

Model 1, Cox proportional hazard model, adjusted for age (continuous), sex (male/female).

Model 2, model 1 plus race (White/Mixed /Asian or Asian British/Black or Black British), alcohol consumption (never/previous/current/missing), smoking status (never/previous/current/missing), body mass index (<25 kg/m^2^/25 to 29.9 kg/m^2^/ ≥30 kg/m^2^/ missing), education level (College or University degree, A/AS-level, O-level/GCSE, CSE, NVQ/HND/HNC, other qualifications, none), Townsend Deprivation index (continuous), UK Biobank assessment center, physical activity (continuous, MET-min/week), diabetes at baseline (yes/no), and cardiovascular disease at baseline (yes/no).

PM_2.5_, fine particulate matter with diameter ≤2.5 μm; PM_2.5-10_, particulate matter with diameter between 2.5 μm and 10 μm; PM_10_, particulate matter with diameter ≤10 μm; NO_2_, nitrogen dioxide; NO_x_, nitrogen oxides.

**Table S4.** Subgroup analysis for the association of coronary artery disease with per 10 μg/m^3^ increase in air pollutants by specific characteristics.

| Subgroups | PM_2.5_ | | | PM_10_ | | | NO_2_ | | | NO_x_ | | |
| --- | --- | --- | --- | --- | --- | --- | --- | --- | --- | --- | --- | --- |
|  | HR (95% CI) | *P^*^* | *P^**^* | HR (95% CI) | *P^*^* | *P^**^* | HR (95% CI) | *P^*^* | *P^**^* | HR (95% CI) | *P^*^* | *P^**^* |
| Age |  |  | 0.391 |  |  | 0.556 |  |  | 1.000 |  |  | 1.000 |
| <60 | 1.04 (0.82, 1.32) | 0.744 |  | 1.07 (0.94, 1.22) | 0.313 |  | 1.02 (0.99, 1.06) | 0.269 |  | 1.01 (1.00, 1.02) | 0.261 |  |
| >=60 | 1.18 (0.99, 1.39) | 0.062 |  | 1.02 (0.93, 1.11) | 0.743 |  | 1.02 (0.99, 1.04) | 0.135 |  | 1.01 (1.00, 1.02) | 0.234 |  |
| Sex |  |  | 0.066 |  |  | 0.429 |  |  | 0.097 |  |  | 0.030 |
| Female | 1.52 (1.20, 1.94) | 0.001 |  | 1.10 (0.96, 1.25) | 0.163 |  | 1.06 (1.02, 1.10) | 0.001 |  | 1.03 (1.01, 1.04) | 0.001 |  |
| Male | 1.13 (0.96, 1.34) | 0.145 |  | 1.03 (0.94, 1.13) | 0.552 |  | 1.02 (0.99, 1.04) | 0.142 |  | 1.01 (1.00, 1.02) | 0.259 |  |
| BMI |  |  | 0.641 |  |  | 0.577 |  |  | 0.507 |  |  | 0.594 |
| Normal | 1.11 (0.82, 1.51) | 0.491 |  | 1.11 (0.94, 1.31) | 0.221 |  | 1.01 (0.96, 1.05) | 0.704 |  | 1.01 (0.99, 1.03) | 0.492 |  |
| Overweight | 1.26 (1.02, 1.55) | 0.031 |  | 1.01 (0.90, 1.12) | 0.917 |  | 1.04 (1.01, 1.07) | 0.013 |  | 1.02 (1.00, 1.03) | 0.012 |  |
| Obesity | 1.33 (1.05, 1.67) | 0.016 |  | 1.08 (0.96, 1.23) | 0.211 |  | 1.04 (1.01, 1.08) | 0.020 |  | 1.01 (1.00, 1.03) | 0.128 |  |
| Smoke status |  |  | 0.800 |  |  | 0.107 |  |  | 0.617 |  |  | 0.758 |
| Never | 1.17 (0.94, 1.46) | 0.157 |  | 1.07 (0.96, 1.20) | 0.237 |  | 1.02 (0.99, 1.05) | 0.267 |  | 1.01 (1.00, 1.02) | 0.160 |  |
| Previous | 1.25 (1.01, 1.54) | 0.038 |  | 0.98 (0.87, 1.09) | 0.674 |  | 1.04 (1.01, 1.07) | 0.015 |  | 1.01 (1.00, 1.03) | 0.044 |  |
| Current | 1.34 (0.95, 1.87) | 0.091 |  | 1.26 (1.04, 1.53) | 0.020 |  | 1.04 (0.99, 1.10) | 0.088 |  | 1.02 (0.99, 1.04) | 0.165 |  |
| Alcohol drinker status |  |  | 0.662 |  |  | 0.563 |  |  | 0.493 |  |  | 0.331 |
| Never | 1.61 (0.91, 2.87) | 0.104 |  | 1.24 (0.89, 1.71) | 0.201 |  | 1.08 (0.99, 1.17) | 0.084 |  | 1.03 (0.99, 1.07) | 0.111 |  |
| Previous | 1.07 (0.61, 1.88) | 0.818 |  | 0.96 (0.70, 1.32) | 0.815 |  | 1.01 (0.93, 1.09) | 0.895 |  | 0.99 (0.96, 1.03) | 0.688 |  |
| Current | 1.25 (1.08 1.45) | 0.003 |  | 1.05 (0.97, 1.14) | 0.209 |  | 1.03 (1.01, 1.05) | 0.004 |  | 1.01 (1.00, 1.02) | 0.005 |  |

*, P value for cox proportional hazard model, adjusted for age (continuous), sex (male/female), race (White/Mixed /Asian or Asian British/Black or Black British), alcohol consumption (never/previous/current/missing), smoking status (never/previous/current/missing), body mass index (<25 kg/m^2^/25 to 29.9 kg/m^2^/ ≥30 kg/m^2^/ missing), , education level (College or University degree, A/AS-level, O-level/GCSE, CSE, NVQ/HND/HNC, other qualifications, none), UK Biobank assessment center, physical activity (continuous, MET-min/week), diabetes at baseline (yes/no), and cardiovascular disease at baseline (yes/no). The stratified factor in each stratum was excluded.

**, P value for interaction analysis between subgroups and air pollutants.

PM_2.5_, fine particulate matter with diameter ≤2.5 μm; HR, hazard ratio; CI, confidence interval; BMI, body mass index.

**Table S5.** Additive joint interaction for included air pollutant exposure and genetic categories on the incident coronary artery disease after excluding participants with a follow-up time of less than 2 years in the UK Biobank.

| Air pollution | CAD PRS (tertiles)^#^ | | | | |
| --- | --- | --- | --- | --- | --- |
|  | Intermediate^†^ | |  | High^†^ | |
|  | RERI (95% CI) | AP (95% CI) |  | RERI (95% CI) | AP (95% CI) |
| PM_2.5_ | 0.24 (0.18, 0.30) | 0.26 (0.19, 0.32) |  | 0.14 (0.06, 0.21) | 0.10 (0.05, 0.16) |
| PM_10_ | 0.24 (0.18, 0.31) | 0.26 (0.20, 0.33) |  | 0.18 (0.10, 0.25) | 0.14 (0.08, 0.19) |
| NO_2_ | 0.29 (0.23, 0.35) | 0.30 (0.24, 0.36) |  | 0.16 (0.09, 0.24) | 0.12 (0.07, 0.18) |
| NO_x_ | 0.32 (0.25, 0.38) | 0.33 (0.27, 0.39) |  | 0.17 (0.10, 0.25) | 0.13 (0.08, 0.19) |

RERI, relative excess risk due to interaction; AP, attributable proportion due to interaction; CI, confidence interval; PRS, polygenic risk score; CAD, coronary artery disease; PM_2.5_, fine particulate matter with diameter ≤2.5 μm; PM_10_, particulate matter with diameter ≤10 μm; NO_x_, nitrogen oxides; NO_2_, nitrogen dioxide.

Adjusted for age (continuous), sex (male/female), alcohol consumption (never, previous, current, missing), smoking status (never, previous, current, missing), body mass index (<25 kg/m^2^, 25 to 29.9 kg/m^2^, ≥30 kg/m^2^, missing), , education level (College or University degree, A/AS-level, O-level/GCSE, CSE, NVQ/HND/HNC, other qualifications, none), UK Biobank assessment center, physical activity (continuous, MET-min/week), diabetes at baseline (yes/no), cardiovascular disease at baseline (yes/no), genotyping batch, and the first 4 genetic principal components.

^†^ Defined by polygenic risk score: low (lowest tertiles), intermediate (second tertiles) and high (highest tertiles).

^#^ To estimate RERI and AP, the lower air pollution category and the lowest genetic risk (low PRS) groups were the reference categories.

**Table S6.** The joint association of PM_2.5_ exposure and coronary artery disease PRS with the risk of incident coronary artery disease after excluding participants with a follow-up time of less than 2 years in the UK Biobank.

| Subgroups | | Case/control | HR (95% CI) | *P* |
| --- | --- | --- | --- | --- |
| Low genetic risk | |  |  |  |
|  | Low PM_2.5_ pollution | 1,935/64,301 | Reference | Reference |
|  | High PM_2.5_ pollution | 2,036/60,008 | 1.07 (1.00, 1.14) | 0.039 |
| Intermediate genetic risk | |  |  |  |
|  | Low PM_2.5_ pollution | 2,544/67,467 | 1.26 (1.19, 1.33) | 3.61E-14 |
|  | High PM_2.5_ pollution | 2,604/63,197 | 1.30 (1.22, 1.38) | <2.0E-16 |
| High genetic risk | |  |  |  |
|  | Low PM_2.5_ pollution | 3,131/68,054 | 1.51 (1.43, 1.60) | <2.0E-16 |
|  | High PM_2.5_ pollution | 3,196/63,546 | 1.56 (1.47, 1.65) | <2.0E-16 |

PM_2.5_, fine particulate matter with diameter ≤2.5 μm; PRS, polygenic risk score; HR, hazard ratio; CI, confidence interval.

Cox proportional hazard model, adjusted for age (continuous), sex (male/female), alcohol consumption (never, previous, current, missing), smoking status (never, previous, current, missing), body mass index (<25 kg/m^2^, 25 to 29.9 kg/m^2^, ≥30 kg/m^2^, missing), , education level (College or University degree, A/AS-level, O-level/GCSE, CSE, NVQ/HND/HNC, other qualifications, none), UK Biobank assessment center, physical activity (continuous, MET-min/week), diabetes at baseline (yes/no), cardiovascular disease at baseline (yes/no), genotyping batch, and the first 4 genetic principal components.

**Table S7.** The joint association of PM_10_ exposure and coronary artery disease PRS with the risk of incident coronary artery disease after excluding participants with a follow-up time of less than 2 years in the UK Biobank.

| Subgroups | | Case/control | HR (95% CI) | *P* |
| --- | --- | --- | --- | --- |
| Low genetic risk | |  |  |  |
|  | Low PM_10_ pollution | 2,040/63,952 | Reference | Reference |
|  | High PM_10_ pollution | 1,931/60,357 | 1.00 (0.94, 1.06) | 0.907 |
| Intermediate genetic risk | |  |  |  |
|  | Low PM_10_ pollution | 2,643/66,985 | 1.24 (1.16, 1.30) | 7.25E-15 |
|  | High PM_10_ pollution | 2,505/63,679 | 1.26 (1.19, 1.33) | 4.37E-10 |
| High genetic risk | |  |  |  |
|  | Low PM_10_ pollution | 3,245/67,919 | 1.45 (1.37, 1.54) | <2.0E-16 |
|  | High PM_10_ pollution | 3,08263,681 | 1.52 (1.43, 1.60) | <2.0E-16 |

PM_10_, fine particulate matter with diameter ≤10 μm; PRS, polygenic risk score; HR, hazard ratio; CI, confidence interval.

Cox proportional hazard model, adjusted for age (continuous), sex (male/female), alcohol consumption (never, previous, current, missing), smoking status (never, previous, current, missing), body mass index (<25 kg/m^2^, 25 to 29.9 kg/m^2^, ≥30 kg/m^2^, missing), , education level (College or University degree, A/AS-level, O-level/GCSE, CSE, NVQ/HND/HNC, other qualifications, none), UK Biobank assessment center, physical activity (continuous, MET-min/week), diabetes at baseline (yes/no), cardiovascular disease at baseline (yes/no), genotyping batch, and the first 4 genetic principal components.

**Table S8.** The joint association of NO_2_ exposure and coronary artery disease PRS with the risk of incident coronary artery disease after excluding participants with a follow-up time of less than 2 years in the UK Biobank.

| Subgroups | | Case/control | HR (95% CI) | *P* |
| --- | --- | --- | --- | --- |
| Low genetic risk | |  |  |  |
|  | Low NO_2_ pollution | 2,185/70,000 | Reference | Reference |
|  | High NO_2_ pollution | 2,076/64,283 | 1.04 (0.98, 1.11) | 0.183 |
| Intermediate genetic risk | |  |  |  |
|  | Low NO_2_ pollution | 2,748/73,055 | 1.21 (1.14, 1.28) | 2.37E-10 |
|  | High NO_2_ pollution | 2,751/67,543 | 1.31 (1.24, 1.39) | <2.0E-16 |
| High genetic risk | |  |  |  |
|  | Low NO_2_ pollution | 3,441/73,315 | 1.55 (1.47, 1.64) | <2.0E-16 |
|  | High NO_2_ pollution | 3,342/68,164 | 1.61 (1.52, 1.70) | <2.0E-16 |

NO_2_, nitrogen dioxide; PRS, polygenic risk score; HR, hazard ratio; CI, confidence interval.

Cox proportional hazard model, adjusted for age (continuous), sex (male/female), alcohol consumption (never, previous, current, missing), smoking status (never, previous, current, missing), body mass index (<25 kg/m^2^, 25 to 29.9 kg/m^2^, ≥30 kg/m^2^, missing), , education level (College or University degree, A/AS-level, O-level/GCSE, CSE, NVQ/HND/HNC, other qualifications, none), UK Biobank assessment center, physical activity (continuous, MET-min/week), diabetes at baseline (yes/no), cardiovascular disease at baseline (yes/no), genotyping batch, and the first 4 genetic principal components.

**Table S9.** The joint association of NO_x_ exposure and coronary artery disease PRS with the risk of incident coronary artery disease after excluding participants with a follow-up time of less than 2 years in the UK Biobank.

| Subgroups | | Case/control | HR (95% CI) | *P* |
| --- | --- | --- | --- | --- |
| Low genetic risk | |  |  |  |
|  | Low NO_x_ pollution | 2,155/69,509 | Reference | Reference |
|  | High NO_x_ pollution | 2,106/64,774 | 1.03 (0.97, 1.10) | 0.364 |
| Intermediate genetic risk | |  |  |  |
|  | Low NO_x_ pollution | 2,686/72,718 | 1.20 (1.13, 1.27) | 1.56E-09 |
|  | High NO_x_ pollution | 2,813/67,880 | 1.31 (1.24, 1.39) | <2.0E-16 |
| High genetic risk | |  |  |  |
|  | Low NO_x_ pollution | 3,379/72,910 | 1.48 (1.40, 1.56) | <2.0E-16 |
|  | High NO_x_ pollution | 3,404/68,569 | 1.55 (1.47, 1.64) | <2.0E-16 |

NO_x_, nitrogen oxides; PRS, polygenic risk score; HR, hazard ratio; CI, confidence interval.

Cox proportional hazard model, adjusted for age (continuous), sex (male/female), alcohol consumption (never, previous, current, missing), smoking status (never, previous, current, missing), body mass index (<25 kg/m^2^, 25 to 29.9 kg/m^2^, ≥30 kg/m^2^, missing), , education level (College or University degree, A/AS-level, O-level/GCSE, CSE, NVQ/HND/HNC, other qualifications, none), UK Biobank assessment center, physical activity (continuous, MET-min/week), diabetes at baseline (yes/no), cardiovascular disease at baseline (yes/no), genotyping batch, and the first 4 genetic principal components.
